# Islanding the power grid on the transmission level: less connections for more security

**DOI:** 10.1038/srep34797

**Published:** 2016-10-07

**Authors:** Mario Mureddu, Guido Caldarelli, Alfonso Damiano, Antonio Scala, Hildegard Meyer-Ortmanns

**Affiliations:** 1 Department of Physics and Earth Sciences, Jacobs University Bremen, Germany; 2 Institute for Advanced Studies IMT, 55100 Lucca, Italy; 3 Dipartimento di Ingegneria Elettrica ed Elettronica Universitá di Cagliari, Italy; 4 ISC-CNR UoS “Sapienza”, Piazzale Moro 5, 00185 Roma, Italy; 5 LIMS the London Institute of Mathematical Sciences, Mayfair, London, United Kingdom

## Abstract

Islanding is known as a management procedure of the power system that is implemented at the distribution level to preserve sensible loads from outages and to guarantee the continuity in electricity supply, when a high amount of distributed generation occurs. In this paper we study islanding on the level of the transmission grid and shall show that it is a suitable measure to enhance energy security and grid resilience. We consider the German and Italian transmission grids. We remove links either randomly to mimic random failure events, or according to a topological characteristic, their so-called betweenness centrality, to mimic an intentional attack and test whether the resulting fragments are self-sustainable. We test this option via the tool of optimized DC power flow equations. When transmission lines are removed according to their betweenness centrality, the resulting islands have a higher chance of being dynamically self-sustainable than for a random removal. Less connections may even increase the grid’s stability. These facts should be taken into account in the design of future power grids.

Over the last years, the increasing power production via renewable energy sources (RES) has completely changed the paradigms underlying energy production, transmission and distribution. To manage RES power output, tools from information and communications technology (ICT) such as remote supervisory and control systems like SCADA/EMS algorithms^1^,^2^ were introduced in power grids in order to make them more efficient, stable and reliable; these technologies can monitor and control the power grids both from an economic and operational point of view. They led to the concept of smart grids. Various concepts for smart grids were proposed and tested^3^,^4^,^5^; when the systems under consideration are small and working as localized groups of generation, storage, and load facilities, they are called microgrids^6^,^7^. Microgrids should provide self-sustaining portions of the network to function even when disconnected from the main grid. Due to their limited size, microgrids can occur at the distribution level and represent an important option to improve the reliability of low-voltage networks. At the distribution level microgrids may be considered as the islands of a management procedure, termed islanding^6^,^8^. In general, islanding consists in splitting the electricity network into fragments that are able to self-sustain their internal power demand. It should preserve sensible loads from outages and guarantee the continuity in the electricity supply, when a high amount of distributed generation occurs. Nowadays distribution and transmission system operators perceive this fragmentation often as a kind of “*noise*” that impairs the voltage and frequency management due to its effects on the standard control system. Moreover, due to the computational burden of islanding procedures, the networks that are accessible to simulations, are often limited in size^9^. As we shall see below, it seems nevertheless quite worthwhile to extend islanding measures towards the transmission level.

Islanding as described before is intentional, it is planned. On the other hand, islanding may happen as a result of outages of lines in an unpredictable and uncontrolled way. Particularly in these cases it is perceived as a threat for utility loads due to the loss of control on voltage and frequency^10^. In this paper we want to simulate outages of branches, which were not foreseen; we analyze the chances of the resulting fragments to recover to a self-sustainable unit by adjusting the production within these fragments. As our results will show, these are the first steps towards “smart islanding”. Smart islanding means the design of power grids via the choice of an appropriate network topology and a suitable distribution of generators, which allow various fragmentation such that most (if not all) of the resulting islands remain self-sustainable under the given conditions.

Here we extend the option of islanding to the level of transmission grids. In this case a large interconnected system should be able to fragment itself in critical situations and enhance the system’s reliability and resilience via automatic procedures like self-healing^11^. To date, however, due to technological^10^ and operational limits^9^, the islanding solution is not implemented at the transmission level, so that local access to energy is not easily available in case of large power outages. Thus, our goal is to identify possible self-sustaining islands, both via topological and operative characteristics.

The vulnerability of increasingly larger power systems is at odds with ongoing projects related to the formation of super-grids, i.e. large-size interconnected transmission grids like the European offshore grid^12^,^13^. In fact, despite the great advantages associated with large grids–mainly related to the trade of energy and economical aspects–concerns are raised with respect to their reliability. A recent theoretical study on cascading in coupled systems^14^ shows that joining several power systems in a super-grid could diminish the occurrence of failures in the individual systems, however, at the expense of an increased probability of system-wide failures. Cascade-like failures or catastrophic events can trigger large-size outages^15^, leading to the suggestion to look for a grid size^16^ that is optimal with respect to the competing goals of profitability and reliability. Thus, the concept of islanding applied to transmission systems would offer a way to enable our large, interconnected grids to split into smaller parts in case of critical events.

A possible approach to look at the problem of grids’ robustness and resilience from a new perspective comes from the young field of Network Science. Network science is focused on the analysis of the topological properties of natural systems, studied by means of their corresponding networks, i.e. the set of nodes *V* = {*i*} corresponding to the system’s elements and a set of links *E* = {(*i*, *j*)} ∈ *V* × *V* that represents the connections between the system’s elements^17^. In the case of power grids, the identification of networks, which can represent these systems, is straightforward. Given the grid’s structure, it is natural to identify the system’s buses with the network nodes and the system’s branches with the network links (or edges). The resulting networks can then be studied on the base of the mathematical framework of graph theory, integrated with methods from statistical mechanics^18^,^19^,^20^,^21^. In the last decade topological properties of electric grids were widely investigated, leading to important results in terms of the systems’ reliability and resilience^15^,^22^,^23^,^24^,^25^,^26^. Particularly interesting are the core measures proposed to understand the network behavior in case of failures, being randomly induced or man-made^27^,^28^,^29^.

The aim of topological methods is to show how electricity networks fragment when links are removed, pointing out in particular how the elimination of central links could have a major impact on the network connectivity (for a more detailed definition of centrality see below). In particular, these studies proved how, in real networks, the removal of a small number of highly central nodes could easily lead to a break up, indicating the fragility of power systems in case of aimed, selective attacks. However, as demonstrated in refs 15 and 30, the networks’ topology alone may be misleading about the vulnerability of the real electricity infrastructure, since it does not account for the nonlinear dynamics, assigned to the grid, in particular ignoring dynamical (rather than geometrical) cascading effects. In fact, recent studies investigated the connection between the topology and dynamics, using a simplified version for the dynamics of power grids^15^,^31^. These studies have shown that black-outs can occur abruptly; in particular, in a study of Pahwa *et al*.^15^ it was shown that larger systems are less resilient to cascade failures, signaling the importance of system size for assessing the reliability of power grids with respect to cascade dynamics. In this paper, we combine topological notions from network science^32^,^33^ with a stability analysis of the energy performance of the power grid based on power flow equations^34^,^35^, a classical method from electrical engineering to model the power grids. We want to mimic unforeseen islanding, caused by natural or operator-induced outages of transmission lines that fragment a power grid. It is expected that in general some of its detached portions will still be able to self-sustain their power production as required by the consumption. Therefore we focus on the conditions, under which all fragments can be made self-sustainable. Moreover, a better understanding of the control parameters and configurations, which favor the formation of self-sustainable islands, will ultimately lead to smart islanding as a safety measure for power grids. We first define our fragmentation procedure for the network, followed by a definition of the tools that we use to study the dynamic stability of the power grid or parts of it. Finally we describe the datasets used to analyze the presence of islands upon fragmentation. We then study the effect of fragmentation, in particular upon varying the amount of renewable energy sources. As test cases we use the German and the Italian power grids. Finally we discuss the impact of our results, their possible applications and future developments.

## Methods and Materials

To study a power grid 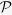, we consider the associated graph *G* = (*V*, *E*), where the set *V* of nodes corresponds to the buses of 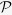, while the set *E* ⊆ *V* × *V* of edges corresponds to the branches of 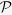. We indicate with *N* = |*V*| the number of buses and with *M* = |*E*| the number of branches.

Our analysis starts from a given stable configuration of the power system on an input data set. The investigation is then based on numerical results in two steps:

- the simulation of the occurrence of failures by either randomly or intentionally removing links,
- the application of the power-flow equations to the resulting power system topology. In this step we evaluate the possibility that its fragmentation can be self-sustainable.

In case of a random failure, these steps can be repeated to accumulate statistics about the occurrence of islands (i.e. self-sustainable fragments) and to estimate the probability that a certain node belongs to a self-sustainable island. We shall vary the so-called penetration *p* of RES into the whole grid: Given the total load *L* and keeping the total generation capacity *C* fixed, we vary the overall RES generation *P*_*RES*_ via the penetration parameter *p* such that *P*_*RES*_ = *p* · *L*, while the conventional power generation *P*_*conv*_ is adapted according to *P*_*conv*_ = *C* − *p* · *L*. Upon variation of p, we only change the ratio of renewable to conventional production, but keep the spatial localization of all generators fixed.

To explore the potential of our procedure, we need model grids based on real data sets where possible; in our case, we will rely on data about the grid topology, generators, renewable sources and power consumption for Italy and Germany.

### Fragmentation into islands

Network science primarily focuses on the mere network topology to develop a systemic understanding of the system under study. Percolation, that is, understanding the fragmentation of a network under disruption of links or nodes, is a classical problem of statistical physics, originally considered for regular or random topologies, but more recently also of network science with arbitrary network topologies. When studying percolation on complex networks it is customary to distinguish the case of random failures, where the choice of discarded network elements is random, from the case of intentional attacks. Intentional attacks typically rank elements according to a certain metric that is a measure for their topological importance. As an example, discarding a node that plays the role of a hub can have a much more dramatic effect than discarding nodes in the periphery of the network^36^.

In discarding branches of the power grid, we consider two cases: we either select an edge at random, or via its rank in terms of its betweenness centrality (*BC*)^37^; since *BC* is a measure for the topological importance of a link in a network, the latter choice mimics an intentional, vicious, strategic attack. In the case of random attacks, we randomly delete a fraction *f* of links and study the number of resulting fragments; since this is a non-deterministic procedure, we repeat it, until we obtain representative average values. In the case of intentional attacks, links are sorted according to their betweenness centrality; for a given fraction *f* of intentional failures, the *f* · *M* edges with the highest values of the metric are deleted. The betweenness centrality *BC* of a link (*i*, *j*) is defined as follows:


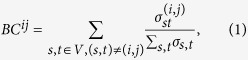


where 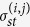 is the number of shortest paths connecting nodes *s* and *t* and passing through the link (*i*, *j*), while *σ*_*s*,*t*_ is the total number of shortest paths between *s* and *t*. Betweenness centrality is a suitable metric for the topological importance, since removing links with high *BC* is an efficient way to split up the network into disconnected fragments^38^. However, its impact on the overall performance of the complex network will depend on the specific dynamics, that is, in our case the power flow in the power grid. To calculate betweenness centrality we use the algorithm proposed by Brandes^39^. In both cases of random and *BC* attacks, the resulting fragments are then separately analyzed to predict, whether they can self-sustain by adapting their production to the power demand. The adapted production is realized via access to the backup capacities of conventional generators, installed on these fragments, as only the conventional generators are easily controllable.

### Power balance of the power grid

In the context of electric engineering a stable performance of the power grid is predicted via solutions of the AC power flow equations^40^, a set of nonlinear equations based on Kirchhoff’s laws. The challenge is to make sure that a fragment of the network can achieve a stable power balance, respecting voltage and frequency constraints, via readjusting the production of conventional generators of the fragment. Since we are first interested only in a qualitative statement about this option, we use a linearized version of the AC power flow, the so-called DC-power flow equations^41^


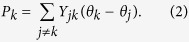


Here, depending on the sign, *P*_*k*_ is the power injected or the load requested from the *k*^*th*^ node, respectively, while *θ*_*k*_ is its phase angle. The matrix *Y* denotes the bus admittance matrix, it can be obtained from *Y*_*e*_, the so-called lines-admittance matrix, where *e* indicating a link *e* = (*i*, *j*), *i*, *j* ∈ {1, …, *M*}. In the absence of mutual impedances between the lines, *Y*_*e*_ is diagonal with diagonal terms being the line impedances *y*_*e*_. *Y* and *Y*_*e*_ are related according to *Y* = *B*^*T*^*Y*_*e*_*B* with *B* the oriented incidence matrix of the system, specifying the buses *k* assigned to each branch *e*,


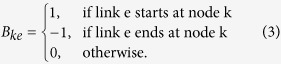


Once Eq. 2 are solved, the power flow *F*_*e*_ in a branch *e* = (*i*, *j*) can be calculated from the phase angles via the incidence matrix as


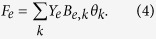


In the context of statistical assessments of the grid balancing, predictions of the DC power flow equations are often only a few percent off those from the full AC equations^42^; for a discussion of the advantages and limitations of the DC power flow as approximation scheme we refer to^43^.

Given the values of the input (power request) and output (power generation), further conditions for a stable grid operation require all branches and buses to operate within their physical constraints. In the simulations, we take the following operative constraints into account:

• Constraints on the power flow in branches: power flow causes dissipation of energy through transmission lines, giving rise to overheating if the developed heat is larger than the losses. For each line *e*, we consider the maximum apparent power 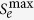 that can flow through such a line. This information is provided by the manufacturer and considered as a parameter of the power system. Therefore the power flow solution is acceptable if for any line:


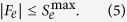


• Global power constraints: In order to maintain a constant frequency over time, the global power balance must be met in real time in the entire system. In particular, the total generation *P*^*prod*^ must balance the power consumption *P*^*load*^ plus the system’s power losses *S*^*losses*^, as stated in Eq. (6)


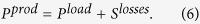


Constraints on the generation output: each power generator at site *k* must supply an amount of injected power *P*_*k*_ that is constrained between minimum 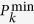 and maximum 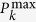 values, given by the electrical and mechanical characteristics of the generator. The power flow solution should therefore satisfy:


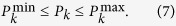


In operating power grids, apart from physical constraints, operators take also economical constraints into account. To implement the latter, it is customary to apply the so-called optimal power flow (OPF) framework^44^,^45^. OPF finds a solution to the generator’s dispatch problem, which is optimal in an economical sense for given physical constraints and generation costs. The generation costs are specified by functions *C*_*k*_(*P*) for each generator *k*, so that the total costs should be minimized:


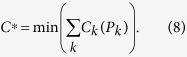


A solution of Eqs (2 and 8) under the constraints (5, 6, 7) will be called a DC OPF. Different solution techniques of DC OPF problems differ in the ratio of precision to computational costs^44^,^45^. We use the MATLAB program MATPOWER^46^, where the MIPS solver determines the DC OPF solution if it exists. Since no economic evaluations have been performed in this study, all the cost functions *C*_*k*_(*P*) of generators have been set to a value, which is an arbitrary constant (in time) and uniform (in space), here chosen as 40$/MWh.

### Datasets of the Italian and German transmission grids

As a concrete application of our procedure for studying smart islanding in power systems, we analyze two datasets, based on real data from the German and Italian transmission systems (as long as the contribution of renewable sources is not artificially increased). The German network was extracted from the UCTE network of ref. 47 and contains an updated version of the network proposed in ref. 48. This dataset describes the full European 380 KV transmission system from an operative point of view, updated in the year 2009. In particular, information on generators’ production and node’s consumption is given, together with the network’s working parameters such as the minimal and maximal power output *P*_min_ and *P*_max_ of the generators, the lines capacities 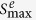 and admittances *B*_*e*_. (Only a few line capacities were not available in the dataset. We estimated their values by available average ratios over all links of the grid of 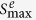 and the calculated power flow *F*_*e*_, that is 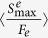). The entire network is considered in a working state as validated by means of a DC power flow simulation. Moreover, each network bus is fully geo-referenced. Power exchange with neighboring countries was implemented as generators or loads on boarder nodes. In addition, we performed a spatial analysis to quantify the amount of energy produced at each node by renewable generators. Thanks to the EnergyMap project site^49^ that maps the position of each RES generator in Germany, we could estimate the total amount of installed renewable generation that exists at each network node by the method explained below.

The Italian 220 kV and 380 KV transmission grids were obtained from the network expansion data from the UCTE network, complemented with a dataset from the website of the Italian transmission system operator TERNA^50^. Data regarding Italian islands like Sicily or Sardinia and 220 KV nodes and branches were added as compared to the original dataset. The dataset includes the geo-referenced position of all 220 kV and 380 kV substations with their electrical characteristics, the geo-referenced position of conventional generators, including their power rates and power ramp limits *P*_min_, *P*_max_, *P*_*rampdown*_, *P*_*rampup*_. The electrical characteristics of the power network such as line impedances and capacities were obtained from^47^ and integrated with^50^. The geo-referenced positions of the installed RES generators are publicly available at the websites of Atlasole^51^ and Atlavento^52^. To estimate the total amount of installed renewable generation, we performed the spatial analysis described below.

In view of the present trend of considerably increasing the amount of RES, it is also important to estimate the impact of RES on smart islanding by artificially varying their amount to larger percentages with respect to the current situation. Remember that we model RES as spatially distributed energy sources, located at the current real places of RES production, at which the amount of renewable energy generation will then be increased, while the conventional production is accordingly decreased. To establish here initial conditions of a working grid, which cannot be taken from real data, we run a new OPF for each value of the penetration parameter p, keeping the total capacity and the total load fixed, and varying the parameters in the algorithm until OPF finds a viable solution. We then start from this solution and remove links either randomly or according to the BC ranking.

Apart from the location we distinguish between renewable and conventional energy production in the way they are used: for balancing the changes in the load on islands after fragmentation we use only conventional sources; this way we take into account that RES may not (yet) be available or tunable as needed. However, we do not implement the impact of their fluctuations when an increased amount of RES further challenges the network stability via their intermittent behavior. It is beyond the scope of this work to pursue a detailed modeling of the fluctuations associated with RES. What we do model when increasing the percentage of RES production is a more distributed production of energy, distributed over all those places, where already nowadays (a smaller amount of) renewable energy is produced. We estimate the average power production of RES via the methods described below.

### Estimation of RES generation at nodes of the transmission grid

Some European countries like Italy (atlasole.gse.it/atlasole/) or Germany (http://www.energymap.info/) published the information about the geographical and technical characteristics of all their installed RES generators. Of particular importance is the information about all RES generators’ nominal power, power production during the last years, technology and geographic position. Under a few assumptions that we shall specify below, we can estimate the amount of installed RES per transmission node, for each specific RES technology. In the following, we describe at the example of the German power system how we assigned RES generation to each node of the system. Along the same steps we proceeded for the Italian power system, with the only difference that the RES generation was associated only with 220 KV nodes.

**Figure 1:**
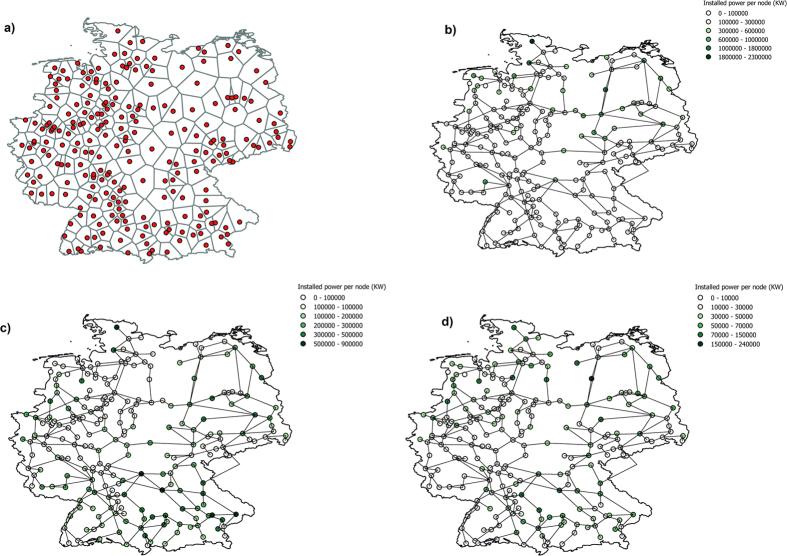
Four maps representing the Voronoi tessellation of the German power system, where red nodes indicate transmission nodes (**a**), the amount of installed wind (**b**), photovoltaics PV (**c**) and biomass generation (**d**) in Germany, for each node of the 380 KV grid. The figure has been produced using the QGIS software^53^.

For each RES generator, the database contains information on the date of installation, the postcode of its geographical location, a registration code, the type of technology (solar, wind, biomass, hydro), the nominal power (in KW), the amount of KWh produced in 2013, the amount of KWh/year produced on average during their lifetime, their geographic position, given in terms of lon/lat with a precision of 0.01 degrees, the responsible TSOs (transmission system operators) and DSOs (distribution system operators). It is hard to assign RES generators to nodes uniquely, because the direction of their supplied power depends on the status of the full system and its power flows, and in principle can vary from time to time. Also a complete knowledge of the entire power system is not available, so that the assignment is at best a good approximation. We assumed that each RES generator supplies its power to the geographically nearest node, and treat this power as a negative load. In general RES generators are too small to be treated as ideal voltage generators, they simply cause a net drop in the nodes’ load on a transmission level. The assignment based on the geographical information was performed by using GIS (Geographical Information Systems) such as QGIS^53^. The area of Germany was first divided into 230 zones, each of them corresponding to an internal node, as shown in Fig. 1(a). These zones were obtained by means of a Voronoi analysis. A Voronoi analysis takes as input a set of points *i*, (here the transmission nodes in Germany), distributed in a territory, and produces a partition in terms of areas *A*_*i*_, each of them corresponding to the area closer to the point *i* than to any other. From the coordinates of the German RES generators we determine the Voronoi area of the transmission node, to which the RES generator belongs to.

As a next step all the installed power in each node is summed up separately for each type of the RES technology along with the installed capacities. The result, that is the amount of installed power for each type of generation technology, is shown in Fig. 1. We use these distributions for the RES production in each node. To a first approximation, the RES power production can be distributed according to the installed capacities. However, for a more precise definition of power outputs during different times a day, it would be necessary to perform further analysis based on more detailed technical parameters. In particular, different production/time curves could be taken into account in the estimate of seasonal and daily power output.

## Results

Our analysis allows to compare (i) the role of intentional versus random removal of links and (ii) the percentage of distributed versus conventional power generation on the dynamics of islanding.

### Random versus intentional removal of links

**Figure 2:**
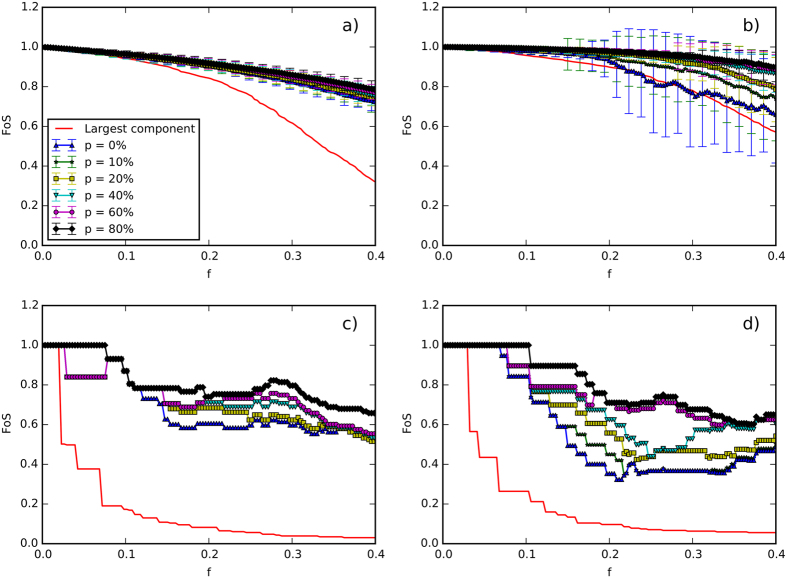
Fraction of nodes *FoS* in self-sustaining islands versus the percentage *f* of removed links. Upper panels: random removal of links; lower panels: link removal based on betweenness centrality (*BC*). Left panels: German grid; right panels: Italian grid. We consider different levels of renewable energy sources *p* (i.e. the percentage of renewable power with respect to conventional power): *p* = 0% (blue upper triangles), *p* = 10% (green stars), *p* = 20% (yellow squares), *p* = 40% (turquoise lower triangles), *p* = 60% (purple circles) and *p* = 80% (black diamonds); the actual amount of renewable sources in Italy and Germany is ~20%. In balancing the power of the islands, the renewable power was kept constant, while only the conventional generation capacity was adapted to balance the power request. The red curves in all panels show the fraction *P*_∞_ of nodes in the largest component (i.e. the largest fragment of the network after link removal). For further explanations see the text.

**Figure 3:**
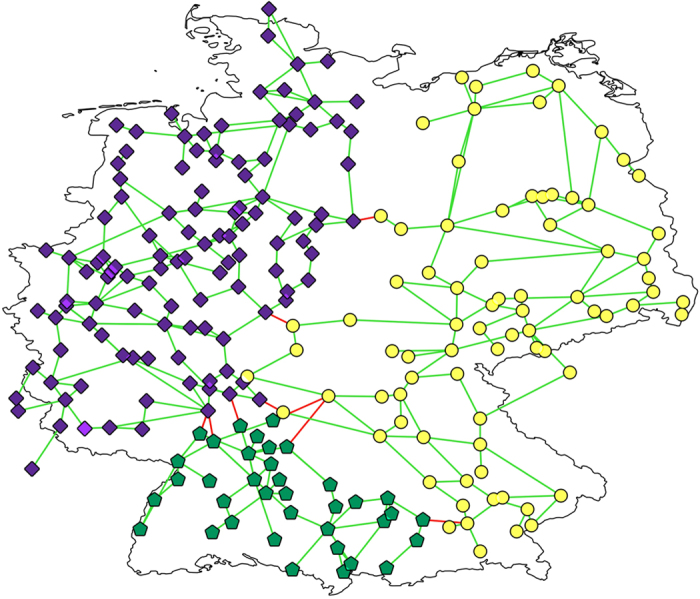
Example of islanding. German transmission network after targeted removal of 8 transmission lines according to their betweenness centrality. Removed links are in red, active ones in green. The three components of the grid would make up a self-sustainable island if we had 40% of renewable sources (roughly twice the actual amount of ~20%), assuming that the fluctuations can be controlled. The figure has been produced using the QGIS software^53^.

In Fig. 2, the upper six curves, almost coalescing in (a), best separated in (d), are obtained for different percentages of distributed versus conventional generation. They show the fraction of working nodes *FoS* as a function of the fraction *f* of removed links, where we do not display values for f = 0.4, because grids with more than 40% removed links are no longer meaningful in view of applications to real grids. The links are removed either randomly (upper panels, (a) for Germany, (b) for Italy), or according to their ranking via betweenness centrality (lower two panels, (c) for Germany, (d) for Italy). For a random removal of links the number of working nodes are averaged over 200 realizations. The error bars refer to the standard deviation, which are large up to 25%, as the randomly selected links may play a very different role in the dynamics of the grid. In case of zero distributed generation, for which the error bars (blue in Fig. 3) are largest, the large errors are caused by fluctuations due to the fact that in the Italian grid even the giant component may not work in certain realizations, differently from the German one. The errors in panels (c,d) are within the numerical accuracy, since the removal of links according to BC as well as the dynamics are deterministic and referring to a fixed (the real) grid topologies.

### Topological role of the giant component

In contrast, the red curves in the four panels of Fig. 2 show the size of the largest component (the giant cluster), measured in terms of its fraction of the total number of nodes. The reason for showing these results in comparison to the other curves is the following. In standard percolation analysis of edge failures, the key quantity is the fraction *P*_∞_ of nodes belonging to the largest connected component (network fragment). Naively one may expect that the largest fragment is more likely to have enough generation power to balance the loads. By definition, the quantity *P*_∞_ is monotonically decreasing with respect to the fraction *f* of erased links; for large systems, *P*_∞_ → 0 at a critical fraction *f*_*C*_ that depends on both, the system topology, and on the order, in which edges are erased^54^,^55^. The red curves in Fig. 2 then show the behavior of *P*_∞_ vs *f* for the Italian and German grid and for random and *BC*-based edge deletion. As expected^36^, the failure of the system for *BC*-based attacks is faster and happens at lower values of *f* with respect to the random failure case. However, *P*_∞_ is a purely topological quantity that is not necessarily related to the dynamical performance and the number of served nodes, as a closer look to the other plots shows.

The number of working nodes in the upper (six) curves is clearly higher than the number of nodes in the giant cluster *P*_∞_. The discrepancy is more pronounced for a removal according to the links’ betweenness centrality than for a random removal. However, in any case the large number of working nodes that remains after the geometric fragmentation shows that a considerable part of the fragments is still working as a power grid. The number of working nodes is even considerably higher, when *P*_∞_ decreases abruptly (as expected^56^). So, from a percolation point of view, it is here not the giant component that is essential for the power grid dynamics. It may even happen that the giant component is dynamically not self-sustainable as seen in panel (b), where the number of working nodes on average is less than the size of the giant component for the Italian grid with zero RES.

### Manifestations of the Braess paradox

Observations, which point in the same direction, are made if we follow the upper six curves as a function of the fraction of removed links. We see non-monotonic behavior occurring for attacks according to the betweenness centrality (c,d), while almost monotonic decrease for random removal (a) and less pronounced non-monotonic for random removal in (b). The fact that after a first decrease the fraction of working nodes may later occasionally increase if further links are removed, is a manifestation of the Braess paradox. In the context of power grids, the Braess paradox means that an additional (transmission) line may decline the performance of the grid, in the extreme case lead to a power outage due to the overloading of certain lines. As such it was identified in connection to power grids^57^. The Braess paradox was first observed in the context of traffic dynamics^58^,^59^, where additional roads may lead to traffic jams. We observe the Braess paradox in the inverse direction: removing lines can increase the number of working nodes on the islands, although in general it is the exception rather than the rule. These results stress the fact that the topology alone is not conclusive for the dynamical grid stability. In contrast to the geometrical role, removing links with high betweenness centrality may improve the dynamical performance due to the creation of self-sustainable islands.

### Role of the giant component in the dynamics

Let us also compare the effects of random versus intentional removal of links (red curves of (a,b) versus (c,d)) on the giant cluster and its dynamical role. From the mere geometric perspective it is expected that the size of the giant cluster decreases more rapidly if links are removed according to their rank with respect to their betweenness centrality. For a link with a high rank its removal cuts off many shortest paths towards another cluster, so that in an extreme case the removal of a single link can reduce the size of the giant component by the size of a whole cluster. From a dynamical point of view, the removal of links according to their betweenness centrality allows more nodes to keep on working while no longer belonging to the giant component as compared to random removal with a larger number of working nodes still belonging to the giant cluster. This tendency is plausible: Consider a case of two clusters, sparsely connected inside the clusters, of similar size, and connected via a single link between the clusters. This link has a high betweenness centrality, as all shortest paths between nodes from one cluster have to pass this link towards nodes of the other cluster. For the power flow dynamics it is very unlikely that this link alone stabilizes the dynamics in each of both clusters. They should be self-sustainable (unless the connecting link has a particularly high capacity), so that its removal should not be of harm for the dynamical performance and allows the option of islanding. In view of smart islanding, eliminating links with high betweenness centrality fragmentizes the grid faster (that is, with less links) into disconnected components than a random elimination. Relative to the fast geometric fragmentation, the number of working nodes is still surprisingly large, even if it is smaller than the working nodes for random removal, after about 10% of the links have been removed. Also the structure of clusters remains widely unchanged if lines with high betweenness centrality are removed. In view of future “designing” smart islanding, an intentional fragmentation according to the betweenness centrality of links may therefore be a good starting point.

### Conventional versus distributed power production

Moreover, in the upper six curves of all four panels, for a given fraction *f* of removed links, the number of working nodes varies, depending on how many generators are conventional and controlled in their power production, while the remaining power production is from RES and spatially distributed over the grid according to the real location data. Starting from the actual current amount of distributed generation in the Italian and German networks, which amounts to 20% (yellow), we varied the amount of distributed generation furthermore from 0% (blue) to 40% (turquoise), 60% (purple) and 80% (black) of the expected total load that is kept fixed, while the percentage of conventional production is accordingly changed. The tendency is obvious. The higher the amount of distributed RES generation, the larger the number of working nodes for a fixed partition of the grid. For example, an increase of distributed generation from 0% to 40% of the system’s load can increase the number of working nodes by up to 30%. This is plausible, as more distributed generation helps in avoiding an overload of links. So these numbers indicate a strong favor for distributed rather than spatially focussed conventional generation as long as the intermittency is neglected. Therefore, the numbers should be taken with care, as the strong fluctuations due to RES are not taken into account. Nevertheless, these results may serve as basis for later analyzing the impact of fluctuations.

### Visualization of islands

**Figure 4:**
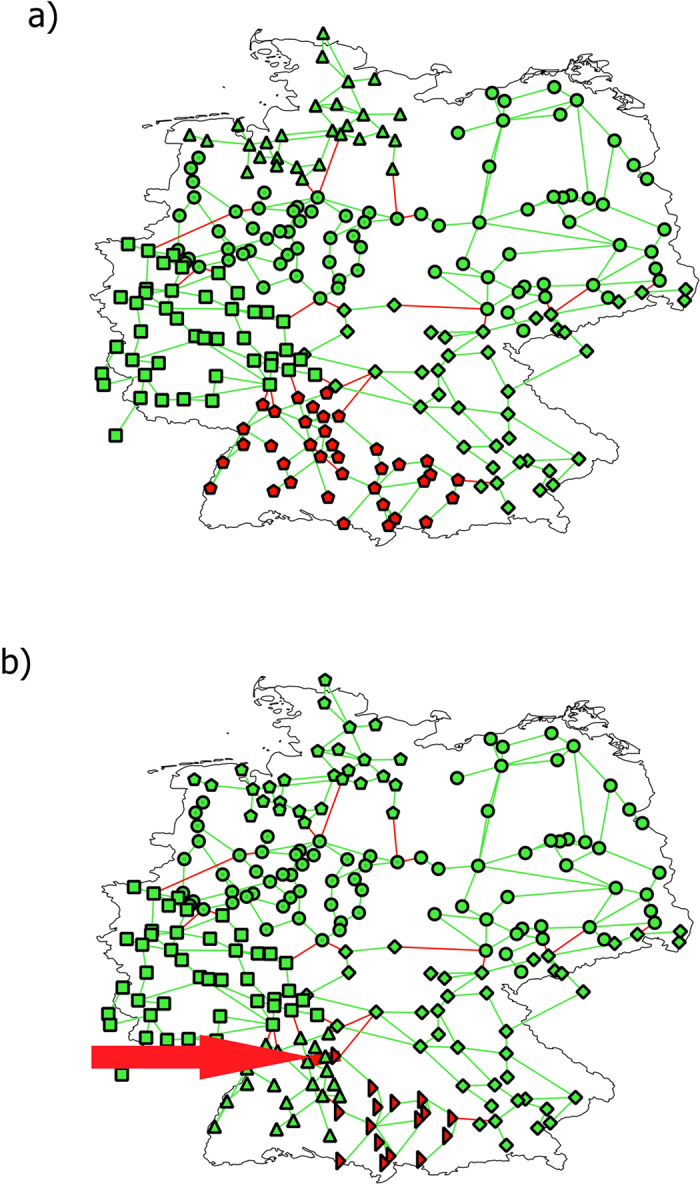
Example of the Braess paradox in case of islanding upon link removals. Upper panel and lower panel show the German grid after removing 23 (**a**) and 24 (**b**) transmission lines according to their betweenness centrality. The different islands of the grids are indicated via different symbols; working nodes and links are green, inactive nodes and removed links are red. The removal of a single link, the 24^*th*^ one, pointed at with the red arrow, increases the number of working nodes by splitting the non-working cluster in the south-west of (**a**) into one working (green symbols) and one non-working (red symbols) in the south west of (**b**). Only 80% RES in the non-working cluster of (**b**) would allow the cluster to become a self-sustaining island. The figure has been produced using the QGIS software^53^.

Since the nodes of Italian and German networks are geo-referenced, it is instructive to actually geographically visualize the islands. For example, after a removal of 8 links, selected according to their betweenness centrality for the German grid in Fig. 3, the grid splits up into three islands, indicated by different colors (along with different shapes for being easier distinguishable in a black-white printout) of nodes. Red links are those, which were removed, green ones are still active. More refined partitions, for example obtained after the removal of 20 and 27 links, lead to partitions with five and seven islands, respectively, not displayed here. One island in the south-west of Germany could be made self-sustainable only by increasing the distributed RES generation to 80% (Fig. 4). Alternatively, the same island could be cured by removing a single link (fat red in Fig. 4), which splits the island into two clusters, the left one (green triangles) becoming self-sustainable for the lower amount of 10% RES, the right one (red rotated triangles) would need 80% RES, to become self-sustainable. This clearly illustrates how the removal of a single line can be a viable way of curing the grid.

## Conclusions and Outlook

In this paper we combined a topology-based approach from network science with energy balancing of the power grid to estimate the grid stability under the outage of transmission lines. We have shown that even if the power grid gets fragmented under an outage, many of the resulting fragments may be self-sustainable if the production within the fragments is readjusted. If this compensation is not sufficient or not feasible within the existing margins to cover the total load, the fraction of distributed generation may be increased as compared to the fraction of conventional production. This means, the higher the percentage of renewable energy sources, the larger is the probability that a fragment is self-sustainable if the fluctuations of RES can be controlled. One option for their control may be distributed storage. Therefore RES are not only ecologically beneficial, but may enhance the power grid security. Smart islanding at the level of the transmission system may therefore mitigate the impact of large blackouts.

Moreover we have shown that a selective removal of transmission lines can be more efficient than their random removal to fragmentize the large grid into dynamically working islands. This result confirms previous warnings that statements on the grid stability should not be based on mere geometric arguments, claiming that attacks according to the betweenness centrality of links are always more harmful than random removals. We have seen the option for the opposite to happen. Less connections can lead to more security, although in general such a manifestation of the Braess paradox will be the exception rather than the rule.

In future work one may further increase the network security by measures taken in advance. Designing a new power grid, it would be constructed as a set of islands, connected by branches, such that a cutout of the branches would leave the islands fully self-sustained. To make them self-sustained, the production should be accordingly adapted or even redistributed. As to existing grids, emergency plans for automatic self-healing may be implemented, based on the idea of smart islanding: For any possibly endangered (set of) branches one would identify the smallest encapsulating islands within a partition of the overall grid. These islands should serve for isolating the failure. They would be chosen under the constraint that the remaining grid can be kept self-sustainable. This way a cascade-like spreading over the whole grid could be prevented.

The geographical location of the islands and the included numbers for distributed versus conventional power production, which we have presented so far, should be taken with care and checked in future work with respect to the following extensions. The inherent approximations in the linearized power flow equations should be compared with more demanding non-linear versions of the power-flow framework. Moreover, when the amount of distributed generation is varied with respect to conventional generation, the strong fluctuations of RES should be taken into account. In emergency cases the adapted production within the islands would set in with delay. In general, the strong fluctuations of RES counteract the self-sustainable operation, unless the fluctuations are effectively damped or averaged by future installed storage capacities. Since simulations of islanding are computationally costly, particulary in the non-linear version, sub-optimal–but faster–algorithms for the black start of transmission system fragments would be a promising next step for implementing resilience in current and future power grids.

## Additional Information

**How to cite this article**: Mureddu, M. *et al*. Islanding the power grid on the transmission level: less connections for more security. *Sci. Rep*. **6**, 34797; doi: 10.1038/srep34797 (2016).
